# Modification of gastric cancer risk associated with proton pump inhibitors by aspirin after *Helicobacter pylori* eradication

**DOI:** 10.18632/oncotarget.26382

**Published:** 2018-12-11

**Authors:** Ka Shing Cheung, Wai K. Leung

**Affiliations:** ^1^ Department of Medicine, The University of Hong Kong, Queen Mary Hospital, Pok Fu Lam, Hong Kong

**Keywords:** aspirin, PPI, H. pylori, gastric adenocarcinoma, triple therapy

## Abstract

**Aim:**

Although proton pump inhibitors (PPIs) can prevent aspirin-induced upper gastrointestinal bleeding, a clinical dilemma exists as long-term use of PPI may also increase the risk of gastric cancer even after *Helicobacter pylori* (HP) eradication. We aimed to investigate the potential interaction between aspirin and PPIs on GC risk in patients who have HP eradicated.

**Results:**

Of the 63,397 HP eradicated subjects (median follow-up 7.6 years), 153 (0.24%) developed GC. PPIs were associated with a higher GC risk among non-aspirin users (aHR: 3.73, 95% CI:2.11–6.60) but not among aspirin users (aHR: 0.35, 95% CI:0.04–2.74).

**Materials and Methods:**

This is a post-hoc analysis based on a previously published territory-wide retrospective cohort study on the potential risk of PPIs on GC. Adults who had received an outpatient prescription of clarithromycin-based triple therapy for HP between 2003 and 2013 were identified. The adjusted hazard ratio (aHR) of GC with PPIs, stratified according to aspirin use, was calculated by Cox model with propensity score adjustment of other covariates.

**Conclusions:**

The potential harmful effects of PPIs on GC development appear to be limited to non-aspirin users. Co-prescription of PPIs is therefore recommended for HP-eradicated patients who are at risk of aspirin-induced UGIB.

Gastric cancer is the fifth commonest cancer and the third leading cause of cancer-related death [1]. Although *Helicobacter pylori (H. pylori)* infection is one of the major risk factors of gastric cancer [2], *H. pylori* eradication could only reduce the risk of gastric cancer by around 40% [3, 4]. We have previously shown that aspirin, one of the most widely available chemopreventive agents, is associated with a reduced risk of gastric cancer in *H. pylori*-eradicated patients (hazard ratio [HR]: 0.30; 95% confidence interval [CI]: 0.15 – 0.61) [5]. However, aspirin increases the risk of upper gastrointestinal bleeding (UGIB), with an incidence rate of 0.91 cases per 1000 person-years in a recent population-based study from the United Kingdom [6]. It is suggested that aspirin users at high risk of UGIB (e.g. prior history of peptic ulcer disease, advanced age, concomitant usage of ulcerogenic agents) should be co-prescribed with proton pump inhibitors (PPIs) [7]. However, as long-term use of PPIs is associated with an increased gastric cancer risk even after *H. pylori* eradication (HR: 2.44; 95% CI 1.42-4.20) [8], it remains a major clinical dilemma whether PPIs should be co-prescribed with aspirin to reduce the risk of UGIB.

To address this unresolved issue, we have investigated the potential interaction of aspirin with PPIs on the risk of gastric cancer after *H. pylori* eradication, based on our previously described cohort who had received clarithromycin-based triple therapy (*n* = 63,397) [8]. The data were retrieved from the territory-wide electronic medical records, known as Clinical Data Analysis and Reporting System (CDARS), of the Hong Kong Hospital Authority. A significant number of populated-based studies have been published using this electronic healthcare database [9, 10]. We hypothesized that the harmful effect of PPIs (in terms of gastric cancer risk) could be negated by the usage of low-dose aspirin among *H. pylori*-eradicated subjects.

In the primary analysis, we used propensity score (PS) regression adjustment with trimming of the extreme PS strata in the Cox proportional hazards model to derive the adjusted HR of gastric cancer with PPI use. PS was the probability of being given PPIs conditional on other covariates including age of receiving *H. pylori* eradication therapy, sex, smoking, alcohol use, comorbidities (prior peptic ulcer disease, diabetes mellitus, hypertension, dyslipidemia, obesity, ischemic heart disease, atrial fibrillation, congestive heart failure, stroke, chronic renal failure and cirrhosis) and concomitant usage of medications (namely, aspirin, non-steroidal anti-inflammatory drugs, cyclooxygenase-2 inhibitors, statins, metformin, clopidogrel and histamine-2 receptor antagonists). We constructed 20 categories of 5% each for the PS distribution and patients in the first and 20^th^ PS strata were trimmed to further reduce biases. Sensitivity analyses were performed by PS regression adjustment without trimming and a multivariable Cox regression.

The median age of this cohort at the time of receiving *H. pylori* eradication therapy was 54.7 years (IQR: 46.0 - 65.4 years), and 46.5% were men. With a median follow-up of 7.6 years (IQR: 5.1 - 10.3 years) totaling 483,260 person-years, 153 (0.24%) patients developed gastric cancer after receiving *H. pylori* eradication therapy. Table 1 shows the gastric cancer risk with PPI use for the whole cohort and according to the usage of aspirin. For the whole cohort, the PS adjusted HR of gastric cancer with PPI use was 2.44 (95% CI: 1.42 - 4.20). Among non-aspirin users, the harmful effect of PPIs appeared to be even larger (HR 3.73; 95% CI: 2.11 - 6.60). PPI use, however, was not associated with an increase in gastric cancer risk among aspirin users (HR 0.35; 95% CI: 0.04 - 2.74). The test for subgroup difference indicates a significant difference between the association of PPIs with gastric cancer in aspirin and non-aspirin users (*p*-value for interaction: 0.010).

**Table 1:** Association between proton pump inhibitors and gastric cancer for the whole cohort and according to aspirin usage

Whole cohort									
	Multivariable analysis (*n* = 63397, GC = 153)			PS adjustment without trimming (*n* = 63397, GC = 153)			PS adjustment with trimming (*n* = 57057, GC = 139)		
	HR	95% CI	*p*-value	HR	95% CI	*p*-value	HR	95% CI	*p*-value
Non-PPI use	Ref	-	-	Ref	-	-	Ref	-	-
PPI use	2.19	1.31 – 3.66	0.003	2.14	1.27 – 3.58	0.004	2.44	1.42 – 4.20	0.002

Non-aspirin use									
	Multivariable analysis (*n* = 54432, GC = 133)			PS adjustment without trimming (*n* = 54432, GC = 133)			PS adjustment with trimming (*n* = 48988, GC = 115)		
	HR	95% CI	*p*-value	HR	95% CI	*p*-value	HR	95% CI	*p*-value
Non-PPI use	Ref	-	-	Ref	-	-	Ref	-	-
PPI use	3.27	1.93 – 5.53	<0.001	3.38	1.99 – 5.75	<0.001	3.73	2.11 – 6.60	<0.001

Aspirin use									
	Multivariable analysis (*n* = 8965, GC = 20)			PS adjustment without trimming (*n* = 8965, GC = 20)			PS adjustment with trimming (*n* = 8067, GC = 16)		
	HR	95% CI	*p*-value	HR	95% CI	*p*-value	HR	95% CI	*p*-value
Non-PPI use	Ref	-	-	Ref	-	-	Ref	-	-
PPI use	0.53	0.12 – 2.37	0.402	0.52	0.11 – 2.34	0.392	0.35	0.04 – 2.74	0.318

Non-PPI use were defined as less than weekly use of PPIs; PPI use was defined as at least weekly use of PPIs. Abbreviations: PS, propensity score; GC, gastric cancer; HR, hazard ratio; 95% CI, 95% confidence interval; PPI, proton pump inhibitor.

In conclusion, we observed in this cohort of *H. pylori*-eradicated subjects that aspirin use appears to negate the potential harmful effect of PPIs on gastric cancer development. Hence, co-prescription of PPIs should still be recommended in *H. pylori*-eradicated patients at risk of aspirin-related UGIB without the concern of an increased gastric cancer risk.
